# Mixed* Lactobacillus plantarum* Strains Inhibit* Staphylococcus aureus* Induced Inflammation and Ameliorate Intestinal Microflora in Mice

**DOI:** 10.1155/2017/7476467

**Published:** 2017-07-27

**Authors:** Dayong Ren, Shengjie Gong, Jingyan Shu, Jianwei Zhu, Fengjun Rong, Zhenye Zhang, Di Wang, Liangfeng Gao, Tianming Qu, Hongyan Liu, Ping Chen

**Affiliations:** ^1^College of Food Science and Engineering, Jilin Agricultural University, Changchun 130118, China; ^2^Veterinary Science Department, College of Veterinary Medicine, Jilin University, Changchun 130062, China

## Abstract

*Objective*.* Staphylococcus aureus* is an important pathogen that causes intestinal infection. We examined the immunomodulatory function of single and mixed* Lactobacillus plantarum* strains, as well as their impacts on the structure of the microbiome in mice infected with* Staphylococcus aureus*. The experiment was divided into three groups: protection, treatment, and control. Serum IFN-*γ* and IL-4 levels, as well as intestinal sIgA levels, were measured during and 1 week after infection with* Staphylococcus aureus* with and without* Lactobacillus plantarum *treatment. We used 16s rRNA tagged sequencing to analyze microbiome composition. IFN-*γ*/IL-4 ratio decreased significantly from infection to convalescence, especially in the mixed* Lactobacillus plantarum *group. In the mixed* Lactobacillus plantarum* group the secretion of sIgA in the intestine of mice (9.4–9.7 ug/mL) was significantly higher than in the single lactic acid bacteria group. The dominant phyla in mice are* Firmicutes*,* Bacteroidetes*, and* Proteobacteria*. Treatment with mixed lactic acid bacteria increased the anti-inflammatory factor and the secretion of sIgA in the intestine of mice infected with* Staphylococcus aureus* and inhibited inflammation.

## 1. Introduction

*Staphylococcus aureus* is a major cause of human infection worldwide [1], is a common pathogen in nosocomial infections, and is a zoonotic pathogen [2]. The host produces a large quantity of proinflammatory cytokines and immune regulatory factors in response to pathogen invasion, which can cause multiple organ dysfunction syndrome [3] or even death.* S. aureus* can express a variety of exotoxins that attack the host immune system [4]. The ratio of Th1 and Th2 cells is important for normal immune response [5]; meanwhile, in an adaptive immune response IFN-*γ* is a subset of CD4^+^ T helper 1 cells (Th1) that plays an important role in severe inflammation [6] by promoting inflammatory response.* S. aureus* infection can induce the release of a variety of inflammatory factors, such as IFN-*γ* [7].* S. aureus* can also cause intestinal infection. SIgA, which is produced by the intestinal mucosa, can inhibit the invasion of bacteria, can help maintain balance in the body, and has an important protective effect on the intestinal mucosa [8].* S. aureus* secretes IgA hydrolase, which helps to inhibit bacterial adhesion [9].

Many lactic acid bacteria (LAB) are probiotics that regulate the body's immune system and help to prevent infection [10]. Studies have shown that some probiotics promote local immunity and IFN-*γ* production while inhibiting IL-4 secretion associated with allergies [11]. Lactic acid bacteria induce sIgA secretion, inhibit the invasion of pathogenic bacteria, and protect the host intestinal tract [12]. Some probiotics, such as* Lactobacillus plantarum*, are involved in mediating immune function [13] and can enhance the immunological function of immunosuppressed mice infected with* S. aureus*. Pro- and anti-inflammatory cytokines and other stress-related signaling molecules play an important role in maintaining balance of immune and inflammatory responses [14]. LAB induce anti-inflammatory factors, such as IL-4 (Th2 cells), while decreasing the levels of enteric or serum regulatory factors in the late stages of infection. This results in a lower number of* Salmonella* in the feces [12]. LAB can inhibit the growth of* Escherichia coli*,* Proteus vulgaris*,* Enterococcus faecalis*,* Staphylococcus aureus*, and other pathogens under suitable pH conditions; this may be due to lactate production [14, 15].

There have been few studies regarding the ability of LAB to ameliorate* S. aureus* infections. Thus, we screened* Lactobacillus plantarum* strains in vitro for antibacterial effects abilities (data not shown).* L. plantarum* was mixed with other LAB to study the effects on immune-related inflammatory factors, sIgA levels, and microbiome composition in mice infected with* S. aureus*. We also verified their abilities to improve immune function and examined whether changes in the immune system are associated with microbiome composition.

## 2. Materials and Methods

### 2.1. Strains

LAB were extracted from fermented foods. The strains,* Lactobacillus plantarum *T4 and T8, were mixed 1 : 1 to form mixed lactic acid bacteria. The strains were cultured in MRS medium at 37°C for 24 h, centrifuged at 7500 rpm for 2 min, and diluted with 0.9% sterile saline to 10^9^ CFU/mL.* S. aureus* was obtained from the Institute of Food Science and Engineering Toxicology Laboratory at Jilin Agricultural University and was cultured in LB medium at 37°C for 24 h and subsequently diluted to 10^6^ CFU/mL. Cultures were stored at 4°C until use. At the end of the test, the bacteria were heat sterilized.

### 2.2. Animals and Feeding

Five-week-old sterile female Kunming mice, weighing 18–20 g, were purchased from the Changchun Institute of Biological Products Co., Ltd. Adequate water was provided, and the mice have free access to chow. Mice were housed in a quiet and ventilated environment; the temperature was 20 ± 1°C, the humidity was 50% ± 10%, and there was natural light. After 1 week of adaptive feeding, the mice were randomly assigned to cages. There were six mice in each experimental group.

### 2.3. Experimental Groups

The experiment was divided into the protection, treatment, and control groups. The protection group was, respectively, fed with* L. plantarum* T4,* L. plantarum* T8, and mixed LAB for 1 week, followed by 1 week of* Staphylococcus aureus*. The mice in the treatment group were infected with* S. aureus* for 1 week and then intervened with single and mixed lactic acid bacteria. The negative control group was given 0.9% sterile saline instead of bacteria. The positive control group was given* S. aureus *for 1 week.

### 2.4. Pathogen Infection in Mice

*S. aureus* was delivered at a dose of 10 mL/kg. Mice were monitored daily. Initial infection was accompanied by lethargy, a reduction in food and water consumption, hair towering, and excretory adhesions; additionally, some sick mice were curled up in a corner. Continued administration led to subcutaneous pustules, part of the epidermis falling off, tail temperature reduced, mice crowding together and not moving, and perianal swelling. The internal organs of the diseased mice were black, indicating a serious illness in the mice. The mice also had a distinct stench. The control mice did not show any abnormal symptoms.

### 2.5. Determination of Mouse Organ Index

The mice were weighed, and the liver and spleen were weighed immediately after euthanasia. Organ index was calculated as [12]: Liver or spleen index = [weight of liver or spleen (g)/body weight (g)] × 1000.

### 2.6. Cytokine IFN-*γ*, IL-4, and Intestinal sIgA Content

Anesthesia was performed by intraperitoneal injection (200 mg/kg/mouse) of a 20% amobarbital and sterile saline solution (1 : 3 v : v ratio). Eyeballs were removed for blood collection and centrifuged at 3500 rpm for 10 min, and the supernatant was collected. One centimeter of the colon was rinsed with sterile saline solution. The intestinal contents were collected and stored at −80°C until use. Serum IFN-*γ* and IL-4 levels were determined by ELISA using anti-mouse IFN-*γ*, IL-4, and biotinylated secondary antibodies according to the manufacturer's instructions. Intestinal sIgA levels were also measured by ELISA. Detection limits for IFN-*γ*, IL-4, and sIgA ELISA assay, respectively, are 50 pg/mL–1500 pg/mL, 0.5 pg/mL–100 pg/mL, and 200 ng/mL–60000 ng/mL. IFN-*γ*, IL-4, and sIgA ELISA kits were purchased from Shanghai Lang Dun Technology Co.

### 2.7. Analysis of Microbial Structure in Excreta of Mice

Microbial DNA was extracted from mouse feces using an Omega kit according to manufacturer's instructions. Extracted DNA was stored at −20°C until use. Samples were sent to the Shanghai Meji Biomedical Technology Co., Ltd., for sequencing using an Illumina sequencing platform. PCR amplification was carried out using 16s rRNA V3-V4 universal primers with barcodes. The primers were 338F (5′-ACTCCTACGGGAGGCAGCAG-3′) and 806R (5′-GGACTACHVGGGTWTCTAAT-3′).

### 2.8. Data Analysis

All results are expressed as mean ± standard error of the mean (SEM). Statistical analysis was performed using Graphpad Prism 6.0 software. Cytokines were analyzed by two-way ANOVA, and Dunnett's method was used for group comparison. *P* values less than 0.05 were considered significant.

## 3. Results

### 3.1. Changes in Body Weight in Mice

**Figure 1:**
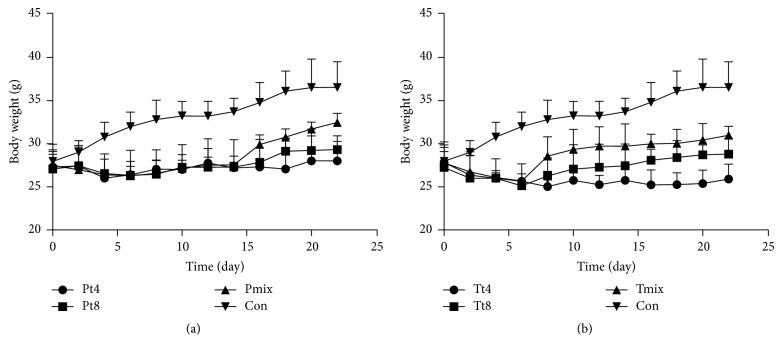
Body weight over time: (a) the protection group (P) containing mice fed* Lactobacillus *spp. (T4, T8, or Mix) for 1 week, followed by a week of* S. aureus* and then normal feeding for a week, and (b) the treatment group (T) containing mice fed with* S. aureus* for 1 week, followed by a week of* Lactobacillus *spp. (T4, T8, or Mix) for 1 week and then normal feeding for a week. The control group (Con) did not receive any processing. Mix: mixed LAB. Data were calculated as mean ± SD (*n* = 6).

The weight of all mice receiving* L. plantarum *increased in the third week (Figure 1(a)), especially the mixed lactic acid bacteria group, which had the most significant increase in body weight (32.0 g) and was closest to the control group (36.5 g). Moreover, the athletic abilities, fecal shape, and fur color of the mice returned to normal. LAB did not significantly alter the body weight of the* S. aureus* infected mice (Figure 1(b)). This indicates that the protection group, especially those treated with mixed LAB, recovered from infection.

### 3.2. Organ Index

The liver index of the mice in the protection group was not significantly different from the control group (48.1, 4.6; Table 1). The mice in the treatment group had liver indices (57.5, 4.7) that were significantly higher than those of the control group (19%, *P* < 0.05). Liver index decreased after 1 week of intervention but was still higher than in the control group (8%, *P* < 0.05). Notably, there is one mouse that died during the* S. aureus *infection in the treatment group.

### 3.3. Serum IFN-*γ* and IL-4 Levels

**Figure 2:**
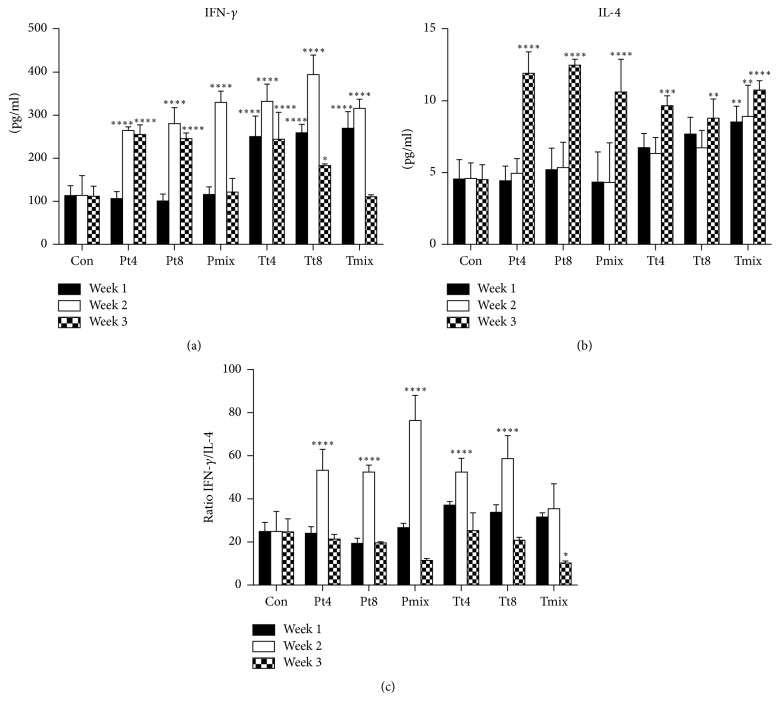
Concentrations and ratios of cytokines in the serum of mice, including serum IFN-*γ* levels (a), serum IL-4 levels (b), and IFN-*γ*/IL-4 ratio (c).^*∗*^ *P* < 0.05, ^*∗∗*^ *P* < 0.01, ^*∗∗∗*^ *P* < 0.001, and ^*∗∗∗∗*^ *P* < 0.0001 versus control. Con: control group, P: protection group, T: treatment group; T4, T8:* Lactobacillus plantarum *strains; Mix: mixed LAB. Data are presented as mean ± SD (*n* = 6).

Compared with the control group, IFN-*γ* was significantly increased (270–320 pg/mL, *P* < 0.001) during infection in both the protection and the treatment groups Figure 2(a). IL-4 did not change significantly (Figure 2(b)). During recovery, IFN-*γ* significantly decreased (110–255 pg/mL, *P* < 0.001) and IL-4 significantly increased (9–12 pg/mL, *P* < 0.001). IFN-*γ*/IL-4 ratio decreased significantly from infection to convalescence, especially in the mixed LAB group (*P* < 0.001) Figure 2(c).

### 3.4. Levels of sIgA in Intestine Contents

**Figure 3:**
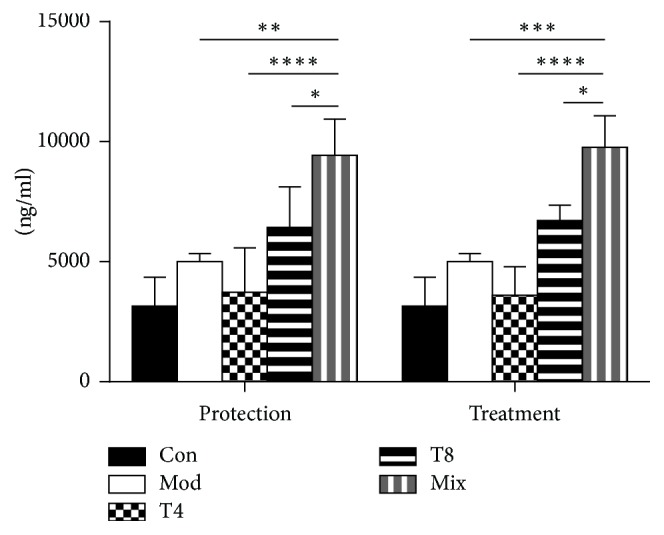
Intestinal sIgA levels. The amount of sIgA was determined by ELISA. Con: control group, Mod: mice infected with* S. aureus *without a week-long intervention with lactic acid bacteria. T4, T8:* L. plantarum *strains, Mix: mixed LAB. ^*∗*^ *P* < 0.05, ^*∗∗*^ *P* < 0.01, ^*∗∗∗*^ *P* < 0.001, and ^*∗∗∗∗*^ *P* < 0.0001 versus mix.

Levels of sIgA in the intestine contents are shown in Figure 3. Compared with the model group, lactic acid bacteria could increase the secretion of sIgA in the intestine of mice infected with* S. aureus*, especially in the mixed lactic acid bacteria group (9400–9700 ng/ml), which was significantly higher than that of the single lactic acid bacteria group (3600–6700 ng/ml, *P* < 0.05), both in the protection group and in the treatment group.

### 3.5. Microbiome Structure

**Figure 4:**
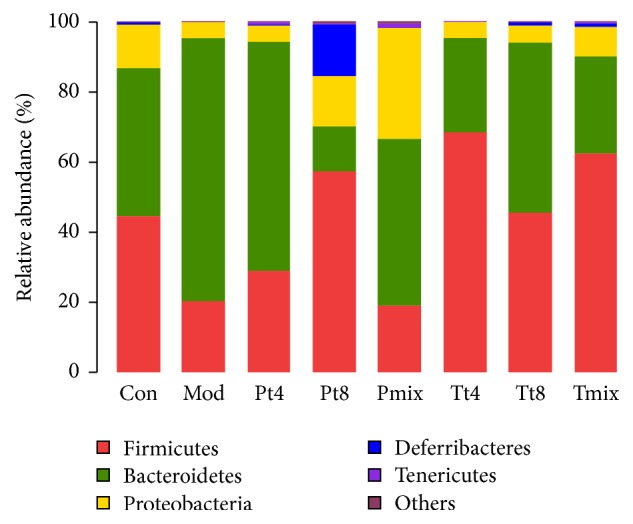
The microbiome composition in different study groups. Each bar represents the average relative abundance of bacterial taxa within a group. Con: control group; Mod: model group; P: protection group; T: treatment group; T4, T8:* L. plantarum *strains; Mix: mixed LAB.

The predominant phyla in mice were* Firmicutes*,* Bacteroidetes*, and* Proteobacteria*, and six different phyla were identified (Figure 4). In the control group, the composition was* Firmicutes* (44.75%),* Bacteroidetes *(42.08%),* Proteobacteria* (12.25%),* Deferribacteres* (0.39%),* Tenericutes *(0.20%), and others (0.33%). In the model group, the composition was* Firmicutes* (20.54%),* Bacteroidetes *(74.60%),* Proteobacteria* (4.55%),* Deferribacteres *(0.03%),* Tenericutes *(0.05%), and others (0.23%).* Bacteroidetes* was the most abundant phylum (47.60%) in the protection group with mixed LAB, which is similar to the levels observed in the control group. In the treatment group, especially mixed LAB, the sum of the relative abundance of* Firmicutes *(62.58%) and* Bacteroidetes *(89.89%; 62.58 and 27.31%, resp.), was similar to that in the control group (86.83%). The results showed that, in the protection group and the treatment group, the flora structure in the mixed lactic acid bacteria group was the closest to the control group.

## 4. Discussion

*S. aureus* is a major human pathogen that causes intestinal infections, and several molecules produced by* S. aureus* cause a strong inflammatory response on the cell surface [4]. After the attack is recognized by TLR2, the corresponding cascade signal transduction is promoted, ultimately leading to the induction of cytokine production and other related immune responses [16].* Lactobacillus* spp. are normal components of human intestinal flora [17] and can regulate the body's immune system [10] to promote local immune prophylaxis and induction of anti-inflammatory factors [11]. In this study,* L. plantarum* (T4 and T8) were screened by in vitro antibacterial test and used to study the protective effects of LAB during* S. aureus *infection.

Earlier studies have shown that mice infected with pathogenic bacteria have a greater loss in body weight and that probiotic feeding-infected mice exhibit minimal loss of body weight [12]. In this study, the lactic acid bacteria mixed group of the protection arm and control group mice exhibited the same trends in weight gain; meanwhile, the mixed LAB group in the treatment arm had no significant changes in body weight. Wan et al. showed that LAB can inhibit infections of the liver, spleen, and other organs in mice [18]. The liver index of the protection group was similar to the control group; meanwhile, the mice infected with* S. aureus* exhibited hepatomegaly. The liver index was decreased by intervention with LAB but was still higher than in the control group. Our results show that LAB can prevent liver enlargement in mice infected with* S. aureus*.

Th1 mediates cellular immunity, which is able to capture intracellular bacteria, viruses, and cancer, inducing the secretion of IFN-*γ* cytokines, a Th2 response to humoral immunity, and IL-4 secretion [19]. Studies have shown that serum IFN-*γ* content is higher in mice following* S. aureus* infection [7]. Activate LAB increase Th1 type IFN-*γ* cytokine secretion in spleen cells of mice infected with* S*.* aureus* while reducing the secretion of Th2 type IL-4 cytokines [11, 20]. In our study, serum IFN-*γ* levels were higher in the infection group compared with the control; meanwhile, the secretion of IL-4 decreased. Kemgang et al. showed that serum IL-4 levels (i.e., Th2 cells) increased in the late stage of LAB intervention [12]. Other studies have shown that IL-4 is the key to the survival and proliferation of T cells, as IL-4 promotes the production of Th2 cells and excessive IL-4 inhibits the production of IFN-*γ* [21]. In this study, the recovery of IFN-*γ* decreased and IL-4 increased. As a result, the ratio of IFN-*γ*/IL-4 in the serum of mice treated with mixed LAB was reduced in the protection and treatment groups between infection and recovery. Our results show that mixed LAB inhibit the production of inflammation in* S. aureus *infected mice.

sIgA is an immune barrier that prevents the adhesion and penetration of toxins, intestinal bacteria, and intestinal epithelial cells. Additionally, slgA inhibits allergenic and pathogenic microorganisms [22, 23] and prevents bacteria from destroying the intestinal mucosa. Jiang et al. showed that sIgA levels in the intestinal tract of infected mice are significantly higher than in controls [24]. In our study, sIgA levels in the intestinal contents of mice treated with LAB were significantly higher in the protection and treatment groups than in the control group, particularly in the mixed LAB arms. This may be due to the promotion of slgA secretion by LAB [12].

There are many microbial species in the intestines.* Firmicutes* are linked to the ability to harvest energy and absorb nutrients from food [25]. Interestingly, cluster analysis of operational taxonomic units showed administration of * L. plantarum* ZDY2013 for 3 weeks significantly increased the abundance of Proteobacteria [26]. In our study, the abundance of Proteobacteria in the intestine of mice treated with mixed LAB in the protection group also increased; meanwhile, the levels of Deferribacteres in the* Lactobacillus plantarum* T8 group were significantly higher than in the other groups. Deferribacteres have not been extensively studied, and their specific function is unknown. Xie et al. showed that oral administration of a probiotic resulted in an important, yet transient, alteration in the small intestinal microbiota that should confer a beneficial effect on the host [26]. Based on our results, mixed LAB have a potentially probiotic effect on the intestinal microbiome of mice infected with* S. aureus*.

## 5. Conclusion

Mixed LAB restored normal growth in mice following* S. aureus* infection, increased levels of the immune-related anti-inflammatory factor IL-4, and promoted secretion of sIgA. These results indicate that mixed LAB could resolve inflammation and promote a healthy microbiome in mice infected with* S. aureus*. This provides evidence for the use of probiotics for treatment of* S. aureus *infections.

## Figures and Tables

**Table 1:** Organ weights of mice in different groups in the first, second, and third weeks of the experiment. ^*∗*^ *P* < 0.05 versus Con; ^*∗∗*^ *P* < 0.01 versus Con.

	Week 1		Week 2		Week 3	
	Liver	Spleen	Liver	Spleen	Liver	Spleen
Con	48.15 ± 0.17	4.61 ± 0.99	48.61 ± 1.99	4.55 ± 1.12	47.87 ± 5.65	4.62 ± 0.41

P						
T4	47.26 ± 0.18	4.54 ± 1.54	48.34 ± 7.54	4.58 ± 1.15	49.09 ± 5.32	5.45 ± 0.88
T8	47.05 ± 1.34	4.61 ± 2.08	46.61 ± 5.28	4.35 ± 1.40	48.82 ± 3.21	4.22 ± 0.81
Mix	48.57 ± 0.42	4.50 ± 1.40	49.50 ± 9.40	3.87 ± 0.23	49.53 ± 8.90	4.22 ± 0.57

T						
T4	56.73 ± 0.79^*∗*^	4.62 ± 1.23	49.62 ± 4.23	4.31 ± 2.02	54.00 ± 5.53	4.59 ± 1.77
T8	58.87 ± 0.38^*∗*^	4.83 ± 2.10	54.97 ± 9.10	4.77 ± 2.02	53.93 ± 6.57	4.58 ± 0.26
Mix	58.43 ± 1.15^*∗∗*^	4.72 ± 0.80	52.06 ± 2.80	4.62 ± 0.78	43.33 ± 9.01	4.35 ± 0.88

Mod	58.18 ± 1.14^*∗∗*^	4.67 ± 1.78	56.18 ± 5.37^*∗*^	4.76 ± 1.83	51.77 ± 4.70	5.06 ± 0.97

The percentage of each animal organ relative to body weight presented as mean ± SD. Significantly different (*P* < 0.05) values in each column are noted by a*∗*. Protection group = P, treatment group = T, and control group = Con. Mix = mixed LAB. Mod = model group. Data is presented as mean ± SD (*n* = 6). In the first week, one mouse died in the treatment T8 group; data is presented as mean ± SD (*n* = 5).
